# A unified resource and configurable model of the synapse proteome and its role in disease

**DOI:** 10.1038/s41598-021-88945-7

**Published:** 2021-05-11

**Authors:** Oksana Sorokina, Colin Mclean, Mike D. R. Croning, Katharina F. Heil, Emilia Wysocka, Xin He, David Sterratt, Seth G. N. Grant, T. Ian Simpson, J. Douglas Armstrong

**Affiliations:** 1 The School of Informatics, University of Edinburgh, Edinburgh, UK; 2 Centre for Clinical Brain Sciences, University of Edinburgh, Edinburgh, UK; 3 Computational Biomedicine Institute (IAS-5 / INM-9), Forschungszentrum Jülich, Jülich, Germany; 4 University of Barcelona, Barcelona, Spain; 5 Simons Initiative for the Developing Brain, University of Edinburgh, Edinburgh, UK; 6 Dementia Research Institute, University of Edinburgh, Edinburgh, UK

**Keywords:** Molecular neuroscience, Protein databases, Data integration

## Abstract

Genes encoding synaptic proteins are highly associated with neuronal disorders many of which show clinical co-morbidity. We integrated 58 published synaptic proteomic datasets that describe over 8000 proteins and combined them with direct protein–protein interactions and functional metadata to build a network resource that reveals the shared and unique protein components that underpin multiple disorders. All the data are provided in a flexible and accessible format to encourage custom use.

## Introduction

At neuronal synapses, the proteomes in presynaptic and postsynaptic compartments form complex and highly dynamic molecular networks. These networks mediate signal transduction and plasticity processes that underpin normal (and abnormal) information processing in the brain. We systematically curated proteomic datasets dating from 2000 to 2020, to produce a comprehensive index of the proteins (and their genes) expressed at the mammalian synapse (see Methods for details). This resulted in 58 papers, which when combined, describe a landscape of 8087 synaptic genes.

The set includes 29 post synaptic proteome (PSP) studies (2000 to 2019) contributing a total of 5560 mouse and human unique gene identifiers; 18 presynaptic studies (2004 to 2020) describe 2772 unique human and mouse gene IDs, and 11 studies that span the whole synaptosome and report 7198 unique genes (Table 1, Supplementary Table 1).

**Table 1:**
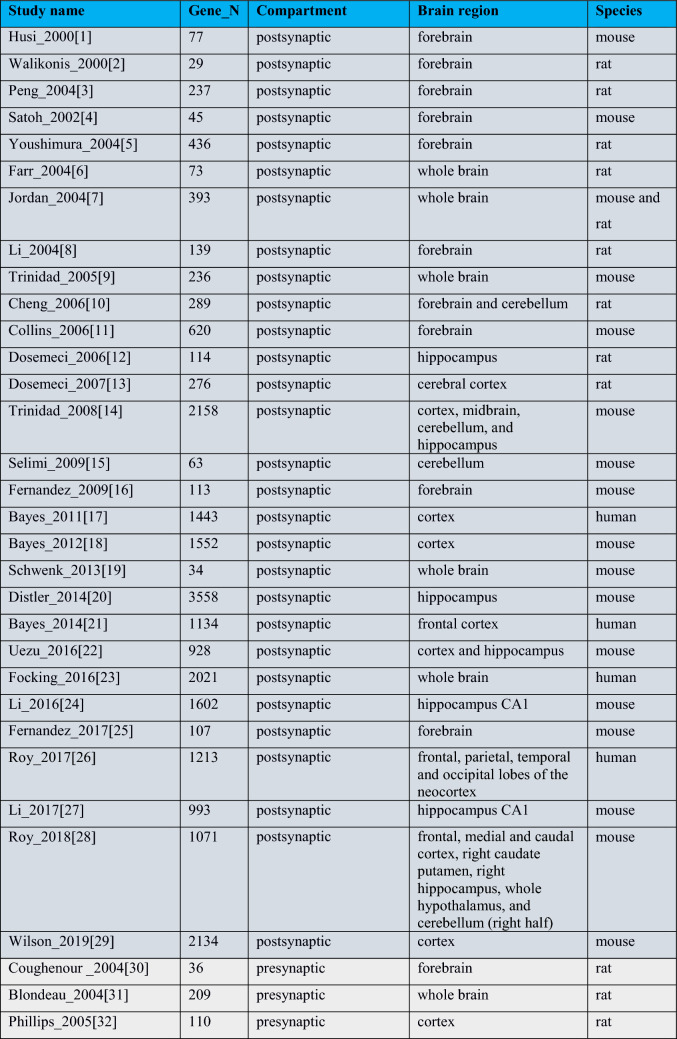

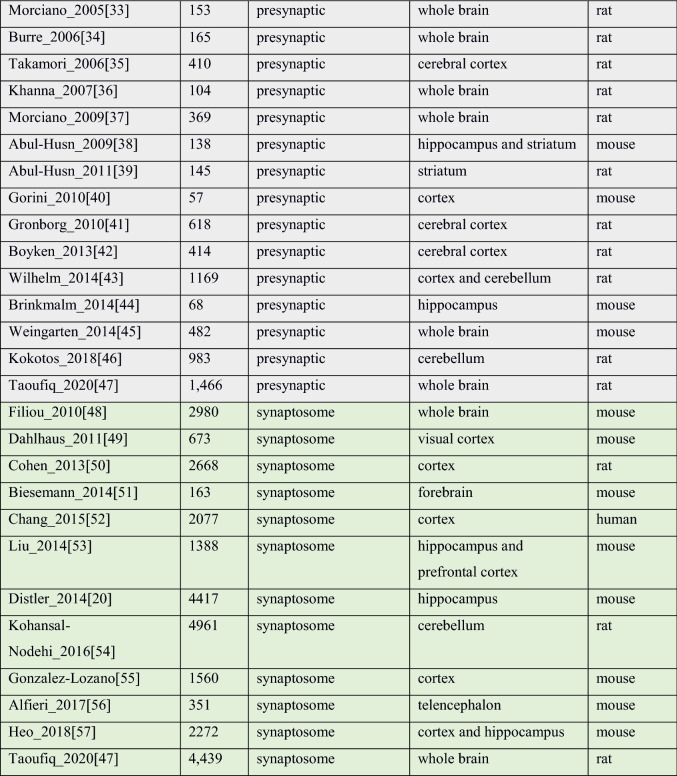
Studies included in the database.

Dark grey corresponds to postsynaptic, light grey—to presynaptic, and green—to synaptosomal studies.

Each study was annotated with relevant metadata including GO function, disease association and cross-ref to SynGo. Orthologues were mapped across human, mouse and rat and each mapped onto stable identifiers (MGI, Entrez and Uniprot).

**Figure 1:**
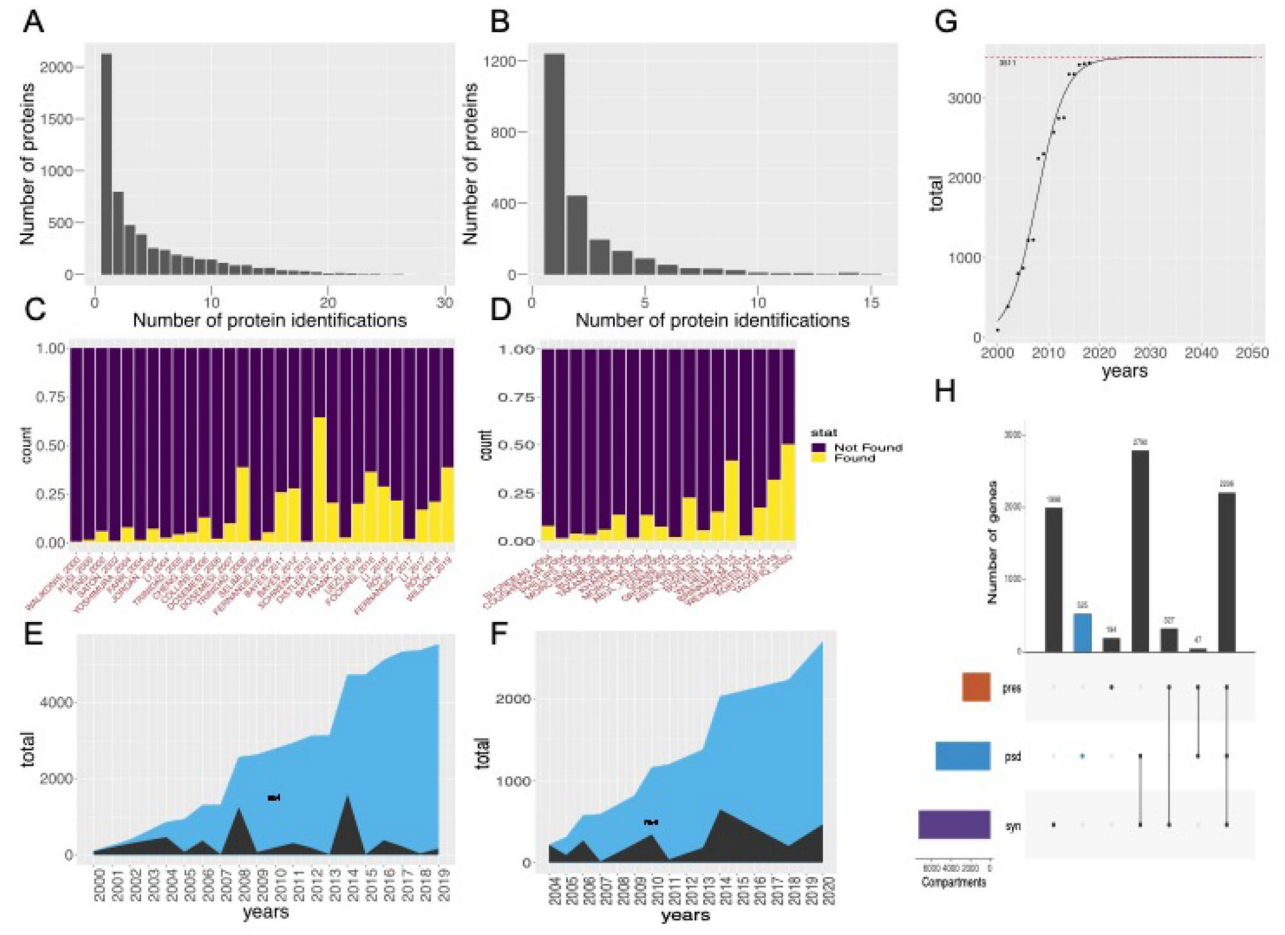
(**A**) Discovery rate of new PSD proteins across 29 postsynaptic studies, where the number of proteins is plotted against the frequency of identification. 2091 PSP proteins have been observed just once. The most frequently found proteins (i.e. detected in 22, or more, studies out of the 29) include very well-known PSD proteins, for example: DLG4 (28/29), CAMK2A (27/29), INA (26/29), SPTBN1, CAMK2B, DLG2, NSF, GRIN2B, GRIN1 (25/29), BIAP2, BSN (24/29) (full list in Supplementary Table 2). (**B**) Discovery rate of new proteins analysed across 18 presynaptic studies. More than half of the proteins in the presynaptic proteome (1251) have been observed just once. The most frequent presynaptic genes include AP2B1, HSPA8, GNAO1, ACTB (15/17), STX1B, ATP6V0A1, STXBP1, ATP1A3, ATP6V1E1, SYT1, GNB1, TUBA1A, VAMP2, NSF, DNM1 (14/17) with full statistics available in Supplementary Table 3. (**C**) Contribution of each of 29 studies to the total number of PSP genes (purple—total number of genes, yellow—identified in this study). Two major jumps in the gross number of proteins identified occur in 2008, when 1249 new proteins were reported by^14^ and in 2014 with 2588 new proteins added by^20^. (**D**) Contribution of each of 18 studies to the total number of presynaptic genes (purple—total number of genes, yellow—identified in this study): two jumps in newly discovered proteins correspond to studies in years 2010 and 2014. (**E**) Accumulation of the new PSP genes (black) compared to the total datasets (blue) over years. (**F**) Accumulation of new presynaptic genes (black) compared to the total datasets (blue) over years. (**G**) Non-linear fit predicting the total size of “consensus” PSP (genes found in two and more studies, 3499) (P = 2.36E−11, residual standard error: 192.7 on 12 degrees of freedom) by year 2023 which, when compared to the current number (3438) indicates that our knowledge on PSP components, based on currently available methodologies, is close to saturation. (**H**) Overlap of three synaptic datasets: presynaptic, postsynaptic and synaptosomal. Bars correspond to the number of unique genes in each compartment and their intersections.

High throughput proteomic techniques are powerful, but they are noisy, and contamination is always a concern. A large number (2091 for PSP and 1434 for presynapse, Fig. 1A,B) of proteins have been observed just once. While single hits may be accounted for lack of sensitivity with low abundance molecules, it could also indicate the presence of false positive components brought in by experimental uncertainty.

The rate of growth with respect to newly discovered proteins for PSP appears to be slowing (Fig. 1C,E) and therefore there is now an opportunity to define a more reliable subset. Following the approach described in^11^, we selected genes found in two or more independent studies to designate the “consensus” PSP. This resulted in 3,438 genes, which is ~ 7 times larger than reported by^11^ and described a subset of synaptic proteins for which have higher confidence. In this subset we observe the increment of new genes per year decreases after 2008 and drops completely after 2014 (Fig. 1C). Based on this, we predict a total number of consensus PSP genes found to be 3499 (Fig. 1G) by year 2023 which, when compared to the current number indicates that our knowledge on PSP components, based on currently available methodologies, is close to saturation.

It is different for the presynaptic compartment, where the recent trend in newly identified genes indicates that saturation has not been achieved yet (Fig. 1D,F). For instance, the latest study by Taoufiq et al.^47^ brought in over 400 new genes to our presynaptic list.

The overlap of proteins found in pre- and post-synaptic datasets, and proteins identified in synaptosomal studies is shown at Fig. 1H and Fig. 1 in Supplementary Methods.

To reconstruct protein–protein interaction (PPI) networks for the pre- and post-synaptic proteomes we used human PPI data filtered for the highest confidence direct and physical interactions from BioGRID^58^, Intact^59^ and DIP^60^. The resulting PSP network contains 4817 nodes and 27,788 edges in the Largest Connected Component (LCC). The presynaptic network is significantly smaller and comprises 2221 nodes and 8678 edges in the LCC.

**Figure 2:**
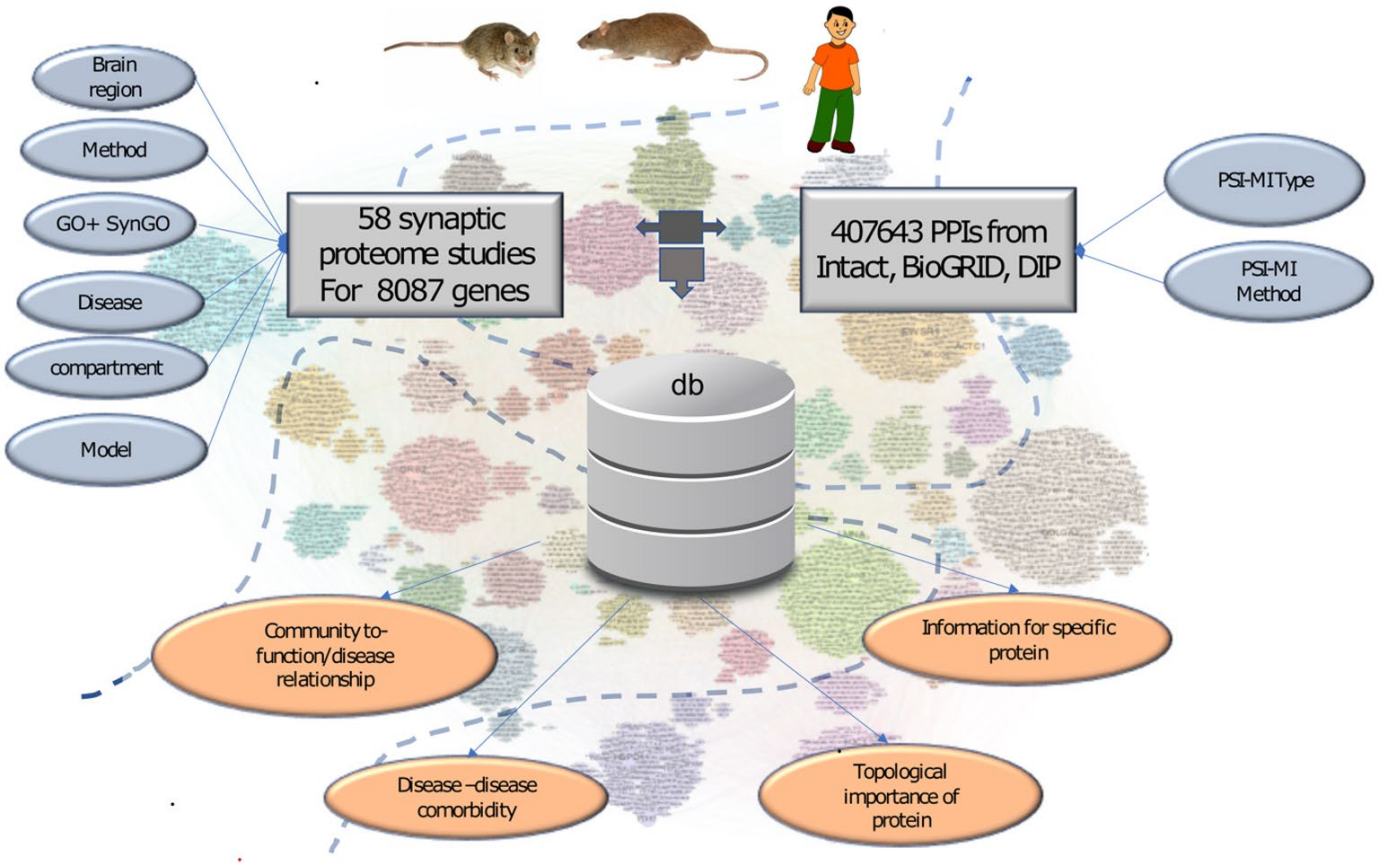
Structure of the SQLite database, which includes 58 synaptic studies covering 8087 unique genes and 407,643 direct protein interactions. Grey ovals on the top show the annotated metadata: left—for nodes/ genes, which include brain region, subcellular compartment, method of extraction, disease and GO function annotation and link to published quantitative models; right—for edges/PPIs, which include PSI-MI type and method. The orange ovals in the bottom illustrate the possible outcomes of the database, including: (1) information for specific protein/gene, and (2) information that could be obtained from PPI network, e.g., protein’s topological importance, community to disease relationship, and disease-disease comorbidity. The database is available as a Supplementary File and from Edinburgh DataShare https://doi.org/10.7488/ds/3017.

The resulting network model is embedded into a SQLite implementation allowing users to derive custom network models based on meta-data including species, disease association, synaptic compartment, brain region, and method of extraction (Fig. 2). The database with manual is available from Supplementary Materials and from Edinburgh DataShare https://doi.org/10.7488/ds/3017, along with a SQLite Studio manual and Rmd file for querying under the R environment, a screencast walk-through demonstrating use-cases can also be found here https://youtu.be/oaW9Yr9AkXM.

The dataset can be used to answer frequent questions such as “What is known about my favourite gene? Is it pre- or postsynaptic? Which brain region was it identified in?”. Beyond that, users can extend these queries to extract custom networks based on bespoke subsets of molecules. Worked examples that are easy to customise are shown in the Supplementary files.

**Figure 3:**
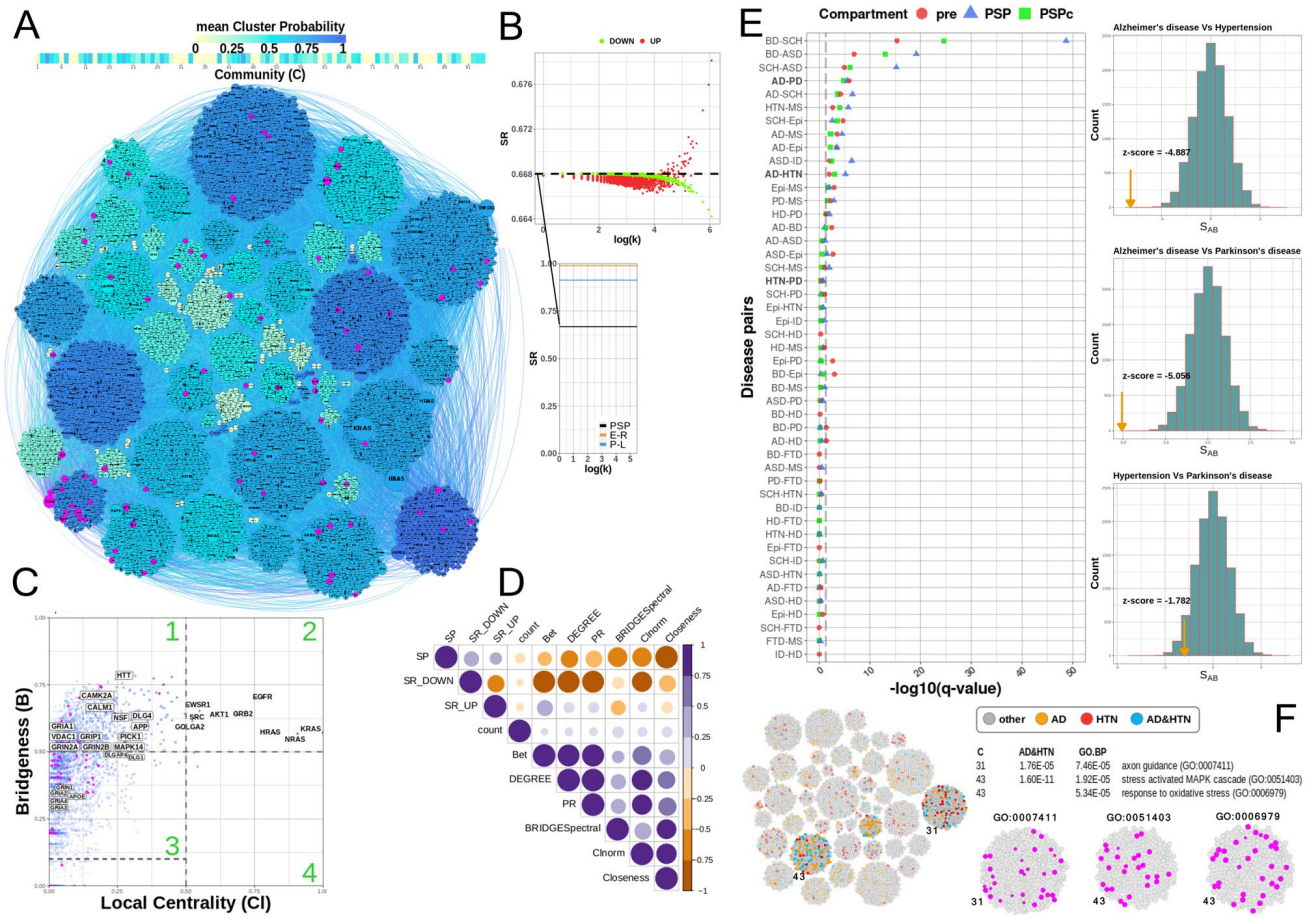
(**A**) Community structure of the PSP network using the Spectral modularity method. Communities are coloured using the average gene-community probability values: bluer coloured a community is, the more probable the genes are of belonging to that community on average. Nodes coloured magenta highlight the core PSD95 interactors^25^, which is also highlighted magenta in the Bridgeness plot in (**C**). (**B**) Graph entropy plots: (main) Global graph entropy rate (SR) plot comparing the structure of the PSP network (0.668) against 1000 randomised Erdos–Renyi (E–R = 0.989 + − 0.0005) and Power–Law (P–L = 0.9127 + − 0.0032, α_PSP_ = 2.41) models of similar size, (Enlarged) Evidence for scale-free structure in PSP network using a perturbation analysis (10], plotted is the SR values after each protein is perturbed through over-expression (SR_UP = red) and under-expression (SR_OWN = green), against the log of the proteins degree,. (**C**) Bridging proteins, estimated using the Spectral clustering algorithm are plotted against semi-local centrality (Methods), allowing their categorisation: Region 1, proteins having a 'global' rather than 'local' influence in the network (also been called bottle-neck bridges, connector or kinless hubs^12^ (DLG4, GRIN2B, CAMK2A, etc.). Region 2, proteins having 'global' and 'local' influence (EGFR, HRAS, NRAS, etc.). Region 3, proteins centred within the community they belong to, but also communicating with a few other specific communities (GRIN1, GRIA2-4). Region 4, proteins with 'local' impact , primarily within one or two communities (local or party hubs^9^. (**D**) Correlation plot for different centrality measures estimated for PSP network.: SP - a protein’s shortest path value, SR_UP-Entropy rate when protein is over expressed, SR_DOWN—entropy rate when protein is under expressed, COUNT - number of protein identifications in the studies, Bet - protein’s betweenness centrality value, Degree—protein degree, PR- Page Rank, BRIDGESpectral —protein Bridgeness value, CNorm - Protein’s local centrality value, Closeness - protein’s closeness value; correlation between SR_UP and Bridgeness indicates that genes with higher Bridgeness values also lower the graphs entropy when active/overexpressed, which allows the signal to pass more freely (Supplementary Table 2). (**E**) left: Disease-disease relationship for presynaptic (red) and PSD full (blue) and PSD consensus (green) interactome. Where significance q-values < 0.05 is delineated by the dashed line. Schizophrenia (SCH), Autistic Spectrum Disorder (ASD), Autistic Disorder (AUT), Bipolar Disorder (BD), Intellectual Disability (ID), Alzheimer disease (AD), Epilepsy Syndrome (Epi), Parkinson's Disease (PD), Frontotemporal Dementia (FTD), Huntington's Disease (HD) and Multiple Sclerosis (MS) are considered; right**:** randomisation studies for disease-disease pairs overlap, yellow arrow shows the measured value of Z-score compared to 10,000 AD-HTN, PD-HTN and AD-PD random models. (**F**) Colocalization of AD and HTN on the PSP network by propagating these gene-disease associations (GDA) through the network using the Belief Propagation DC-SBM algorithm^13^. The colocalization of AD and HTN shared common molecular pathways in communities 31 and 43, which were also found enriched for axon guidance, stress-activated MAPK cascade and response to oxidative stress GO BP terms.

The underlying principle of a systems biology approach is that structural features (pathways and subnetworks) underpin network functionality and given a network, one should be able to extract these features. Clustering algorithms^61,62^ are commonly used to identify local communities within the network under the assumption that shared network topology correlates with shared function (and dysfunction). However, the more important question is how the different communities are organised to enable a controllable flow of signals across the large network. Using the PSP network as example, we identified 1029 “Bridging” proteins as those known to interact locally with neighbours in the network—helping organise function inside communities they belong to^63,64^, and simultaneously influence other communities in the network (Fig. 3A, Methods). Using graph entropy as a compliment means of ranking a protein’s ability to inhibit or enhance information flow^65^, we found that proteins with high Bridgeness value have ability to decrease the entropy of the network thus facilitating the signal transmission (Fig. 3B,D, Methods). Of the 1029 candidate Bridging proteins (see Region 1, Fig. 3C), we found ~ 43%) associated with at least one known synaptopathy and ~ 21% linked to multiple diseases including: APP (AD&Epi&ASD&PD&HTN&MS&FTD), VDAC1 (AD&PD&MS), and MAPK14 (AD&SCH&HD&HTN&MS), which supports the functional/disease importance of “bridging” proteins. Indeed, we found significant overrepresentation for specific diseases, such as AD (P = 3.4 × 10–6), HTN (P = 2.1E−5), HD (P = 5.2E−5), PD (P = 2.6E−3) (Supplementary Table 2).

There are many complex co-morbidities between psychiatric disorders at the population and the genetic level but for most the molecular basis remains elusive. The network perspective can be used to obtain a different view by linking topology and phenotype together. Gene-disease association data is noisy and far from complete, but we can partly compensate by measuring, for each disease, the distance from each protein in the network to its nearest known associated protein, which can be extended to disease pairs^66^ to dissect how these different neurological diseases coalesce at the synapse.

Using PSP (both full and consensus) and presynaptic networks we found clear evidence of network overlap between well-known co-morbid neuro-psychiatric/developmental disorders in both postsynaptic and presynaptic models (q-values shown for PSP/presynaptic networks), including BD-SCH (P = 2.0E−49/4.39E−16), BD-ASD (P = 7.12E−20/1.28E−7), and ASD/SCH (P = 6.17E−16/1.12E−5). Similarly, overlap was observed for common neurodegenerative diseases/conditions AD and PD (P = 3.04E−6/1.32E−6).

We also observed compartment-specific overlaps for Epilepsy with PD (P = 0.53/2.12E−3) and BD (P = 0.54/9.73E−4), which is significant only in the presynaptic network (Fig. 3E).

In both postsynaptic and presynaptic models, we found overlap for Hypertension (HTN) with AD (P = 8.6E−4/1.0E−2, and with MS (P = 8.79E−5/2.12E−3) (Fig. 3E). The AD-HTN link is not, in itself, new but commonly considered as a cardiovascular mechanism with a neurological impact. However, the network view reveals a new potential mechanistic link at the synapse. Although we found significant overlaps between AD-HTN and AD-PD, we did not see evidence for a PD-HTN link (P = 0.17/0.36), which indicates the potential shared mechanistic pathway between AD and HTN, which is different to the pathways shared between AD and PD (Fig. 3E).

To further dissect the potential sharing of pathways between AD and HTN in the PSP network (Fig. 3F), we employed Belief Propagation to propagate these GDA’s through the network’s edges, and a Degree-Corrected Block Model (DC-SBM) to model its effect on network clustering^67^. Under a prior assumption of no correlation between the GDA’s and the network communities, we found evidence for the co-localization of AD and HTN (C = 31 P = 4.69E−5 and C = 43 P = 1.6E−11). Functionally, these communities are enriched for synaptic transmission, axon guidance (C = 31, GO:0007268 = 5.8E−3, GO:0007411 = 7.46E−5), stress activated MAPK cascade and response to oxidative stress (C = 43, GO:0051403 = 1.92E−5, GO:0006979 = 5.34E−5).

The presented synapse proteome dataset is the largest, most complete and up to date and is freely available with lightweight tools to allow anyone to extract relevant subsets. It compliments previously published curated dataset of synaptic genes SynGO^68^, and both resources could be used jointly as we have cross-referenced the common genes. By mirroring the methods used it would be straightforward for any user to add in their own datasets for comparison.

## Supplementary Information

Supplementary Information 1.

Supplementary Information 2.

Supplementary Information 3.

Supplementary Information 4.

Supplementary Information 5.

Supplementary Information 6.

Supplementary Information 7.
