# Author Correction: A unified resource and configurable model of the synapse proteome and its role in disease

**DOI:** 10.1038/s41598-021-95608-0

**Published:** 2021-08-04

**Authors:** Oksana Sorokina, Colin Mclean, Mike D. R. Croning, Katharina F. Heil, Emilia Wysocka, Xin He, David Sterratt, Seth G. N. Grant, T. Ian Simpson, J. Douglas Armstrong

**Affiliations:** 1 The School of Informatics, University of Edinburgh, Edinburgh, UK; 2 Centre for Clinical Brain Sciences, University of Edinburgh, Edinburgh, UK; 3 Computational Biomedicine Institute (IAS‑5/INM‑9), Forschungszentrum Jülich, Jülich, Germany; 4 University of Barcelona, Barcelona, Spain; 5 Simons Initiative for the Developing Brain, University of Edinburgh, Edinburgh, UK; 6 Dementia Research Institute, University of Edinburgh, Edinburgh, UK

Correction to: *Scientific Reports*, 10.1038/s41598-021-88945-7, published online 11 May 2021

The original version of this Article contained an error in the spelling of the author T. Ian Simpson which was incorrectly given as Thomas I. Simpson. The original Article has been corrected.

