# Supplementary material for: A unified resource and configurable model of the synapse proteome and its role in disease
Source: Sci Rep. 2021 May 11;11:9967. doi: 10.1038/s41598-021-88945-7 (PMC8113277; doi:10.1038/s41598-021-88945-7)
Supplement: Supplementary file 2 — Supplementary Information 2. [file 41598_2021_88945_MOESM2_ESM.zip › SQLite Synaptic Proteome database manual_DA.docx]

Synapse Proteomics Database

User Manual

V1.0_draft, April 2021

Contact: Oksana.Sorokina@ed.ac.uk

Table of Contents

1. Overview
2. SQLite Studio
   1. Use case 1: Find the info for a specific protein
   2. Use case 2: Find the info for a list of proteins
   3. Use case 3: Build the PPI network from protein list
   4. Use case 4: Find disease information for my gene of interest
   5. Use case 5: Export results as a flat file

Overview:

The database includes: proteomic and interactomic data with supporting information on compartment, specie and brain region, GO function information for three species: mouse, rat and human, disease annotation for human (based on Human Disease Ontology (HDO)) and GeneToModel table, which links certain synaptic proteins to existing computational models of synaptic plasticity and synaptic signal transduction

The original files are maintained at Eidnburgh Datashare DOI:XXXXXX

The original manuscript describing this dataset is Sorokina et al 2021 (DETAILS TO FOLLOW)

The database contains the following main data tables

- Gene: list of genes including IDs (MGI, Entrez Human and Mouse) and gene names (Human, Mouse, Rat).
- Specie: Tax ID (Human, Mouse, Rat)
- Paper: list of papers with PMID ID, name (in format “FirstAuthor_year”), year of publication
- Location: postsynaptic, presynaptic, synaptosome
- Method: shotgun or IP
- Brain region: list of regions where the samples originate, including hierarchical region structure (Figure1).
- SpecieRegion: species specific projection of regions structure shown on Figure 1
- PPI: human protein-protein interactions combined from BioGRID, Intact and DIP databases, contain information on methods, interaction type ( PSI-MI nomenclature) and PMID info for each of the interactions.
- PaperGene: table links gene to respective papers and the metadata above
- GO: BP, CC and MF GO annotation for Human, Mouse and Rat species
- GOGene: gene to GO association list
- Disease: List of diseases from HDO for Human
- DiseaseGene: genes to disease association list
- GeneToModel: genes with found association with published model of synaptic plasticity

*Figure 1. Heirarchy of brain regions currently annotated in the database.*

Figure 2. Entity Relationship diagram showing the structure of SQLite database for synaptic proteome.

The database scheme (Figure 2) is designated to represent information for specific proteins, list of proteins and PPIs. The information can be manipulated using a variety of tools including SQLite Studio (below) or RStudio (Rmd provided).

SQLite Studio:

See <https://youtu.be/oaW9Yr9AkXM> for a screen recorded walk-though.

1) Download and install SQLite Studio from:

<https://sqlitestudio.pl/index.rvt>

2) Unzip file "synaptic.proteome_SR_20210204.SynGO.db.sqlite.zip" (Supplementary Files)

3) Open SQLite application.

In Menu select "Database" and then "add a database". Choose database type SQLite 3 (default) and brows for " synaptic.proteome_SR_20210204.SynGO.db.sqlite " , press "Ok".

3) On the left-hand side panel you should now see the database, click on it to see the content - You should see 13 tables and 4 Views available for inspection.

Below is shown the table Gene, which contains GeneID information including reference to SynGo database.


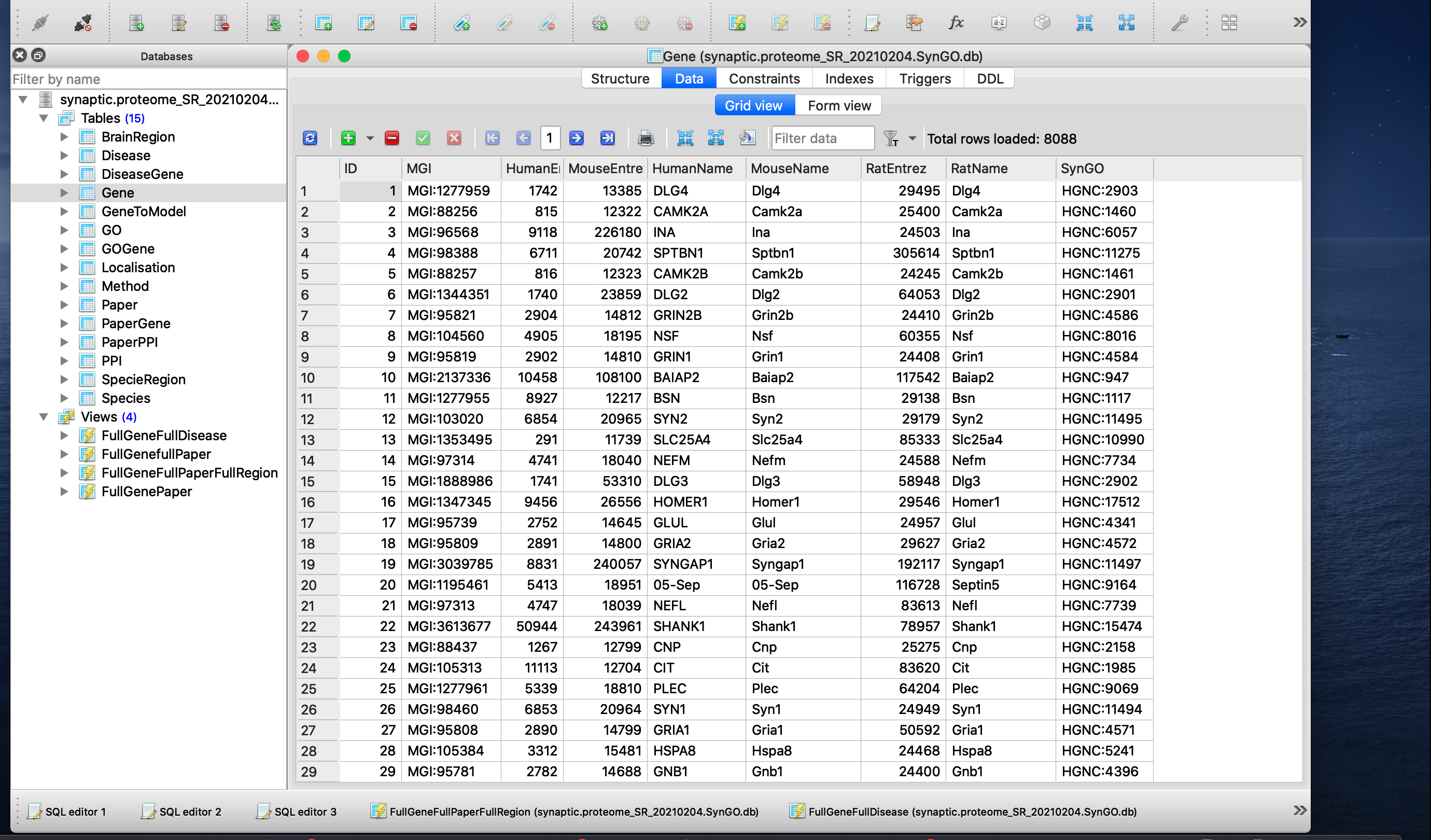


4) Press the "SQL editor 1" at the left bottom to open Query window.

5) Case 1. Find the info for a specific protein:

Insert the following query into the Query window:

SELECT Localisation,

HumanEntrez,

HumanName,

PaperPMID,

Paper,

Year,

BrainRegion

FROM FullGeneFullPaperFullRegion

WHERE HumanName = "SRCIN1"

Press blue triangle on the top left of Editor's menu to execute.


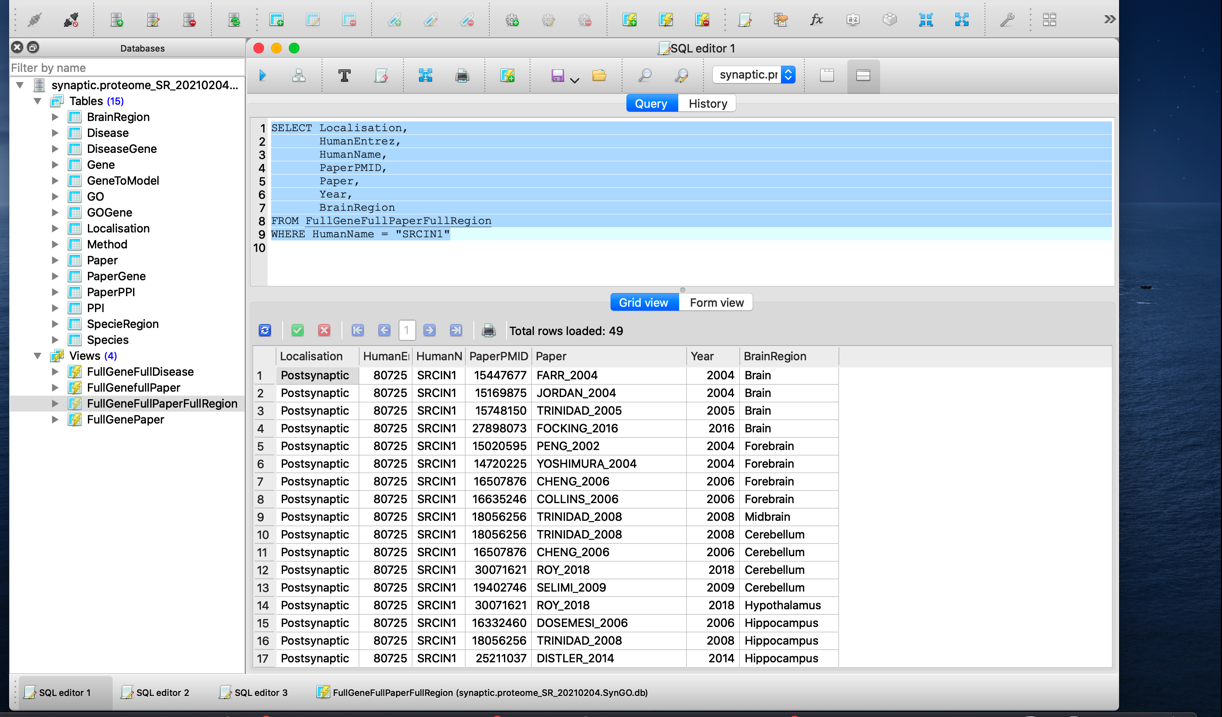


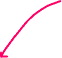


You can modify the query with any protein name of interest.

You can use Entrez ID instead of name, in this case modify query as follows:

SELECT Localisation,

HumanEntrez,

HumanName,

PaperPMID,

Paper,

Year,

BrainRegion

FROM FullGeneFullPaperFullRegion

WHERE HumanEntrez = "1742"


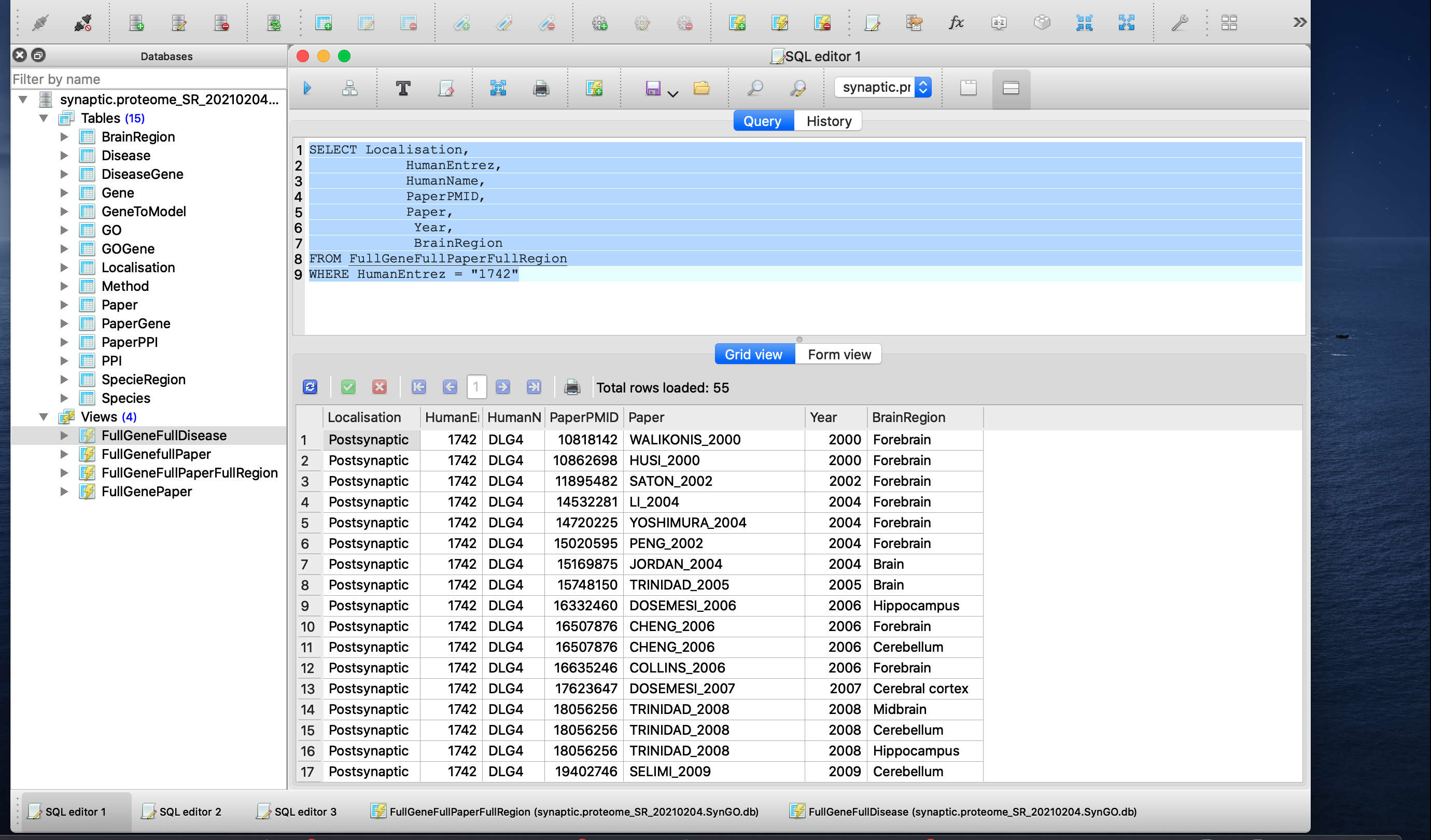


Case 2. Find the info for a list of proteins:

Specify a list of protein names or IDs as follows:

SELECT Localisation,

HumanEntrez,

HumanName,

PaperPMID,

Paper,

Year,

BrainRegion

FROM FullGeneFullPaperFullRegion

WHERE HumanName in ("SRCIN1", "SRC","DLG1")

Case 3. Build the PPI network from protein list.

# Using our database, one could extract PPI map for specific study, brain region or compartment. The example R query for presynaptic protein list and its respective PPI network will look as following:

SELECT DISTINCT f1.HumanEntrez as A,

f2.HumanEntrez as B

FROM PPI

JOIN FullGeneFullPaper f1 ON f1.GeneID = A

JOIN FullGeneFullPaper f2 ON f2.GeneID = B

WHERE f1.Localisation = 'Presynaptic'

AND f2.Localisation = 'Presynaptic'

AND f1.HumanEntrez IS NOT NULL

AND f2.HumanEntrez IS NOT NULL


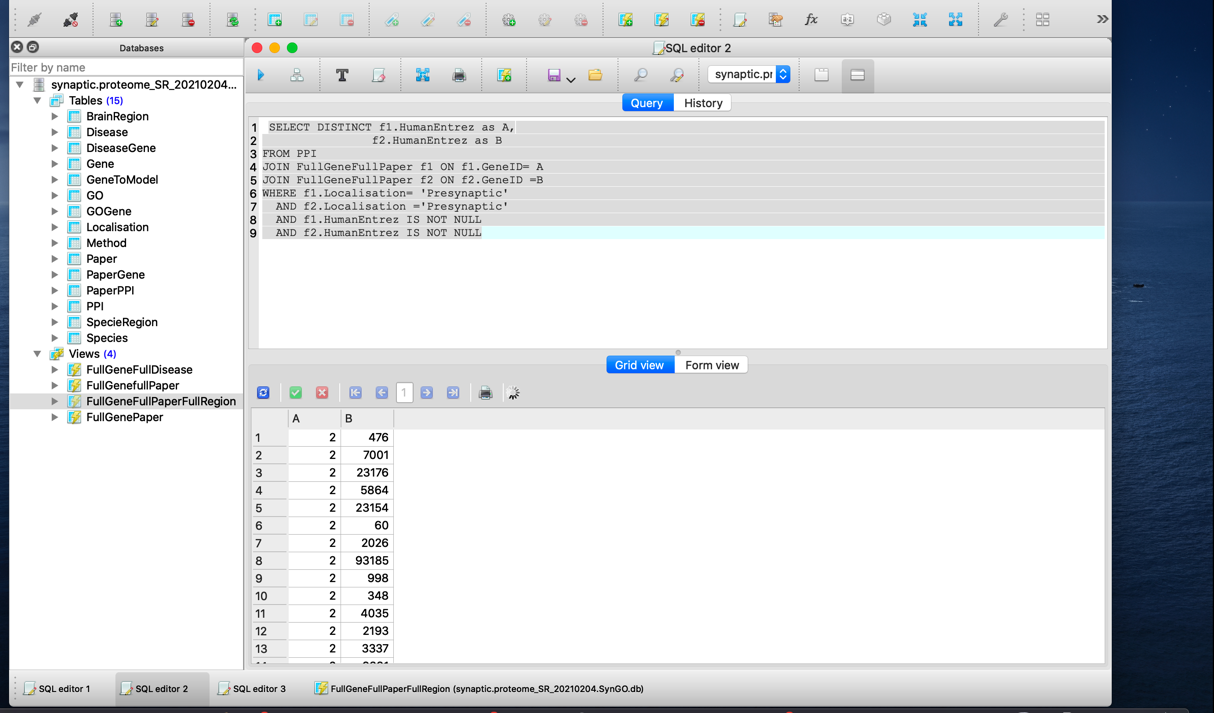


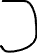


Case 4. Find disease information for my gene of interest.

The View table FullGeneFullDisease contains all the disease association information, that could be queried as follows:

SELECT HDOID,

Description

FROM FullGeneFullDisease

WHERE HumanName = "DLG4"



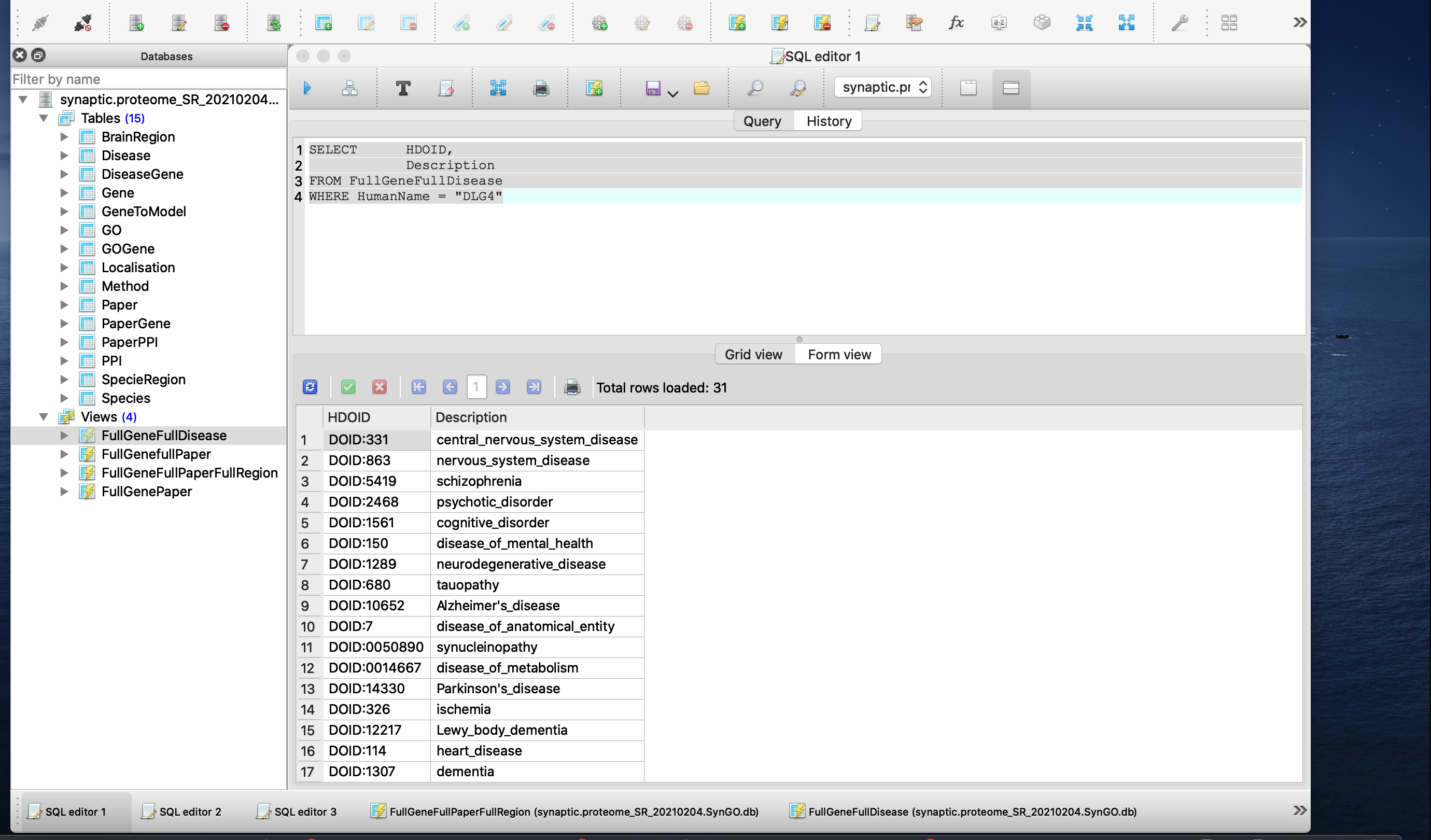


6) To export query results as a table press the Export button, then press Continue, and choose the export format (csv, pdf, etc).


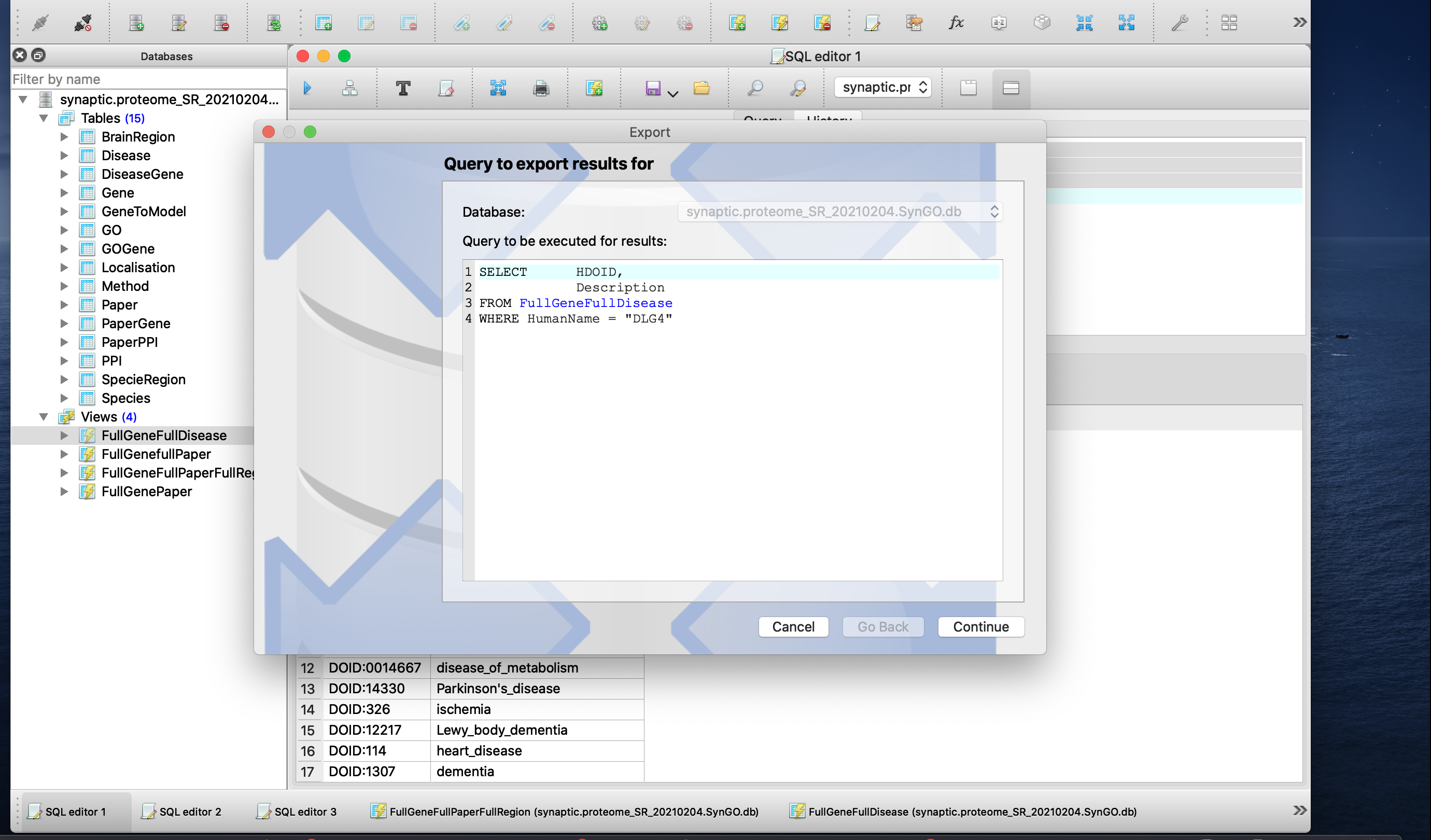


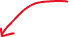


The resulting network structure could be visualised in R, using package Igraph, or it could be saved as csv file and loaded into Cytoscape or Gephi (e.g. Figure 3).

# Figure 3. *Visualisation and analysis example of presynaptic PPI network*
