# Supplementary material for: A unified resource and configurable model of the synapse proteome and its role in disease
Source: Sci Rep. 2021 May 11;11:9967. doi: 10.1038/s41598-021-88945-7 (PMC8113277; doi:10.1038/s41598-021-88945-7)
Supplement: Supplementary file 3 — Supplementary Information 3. [file 41598_2021_88945_MOESM3_ESM.docx]

# A unified resource and configurable model of the synapse proteome and its role in disease

Oksana Sorokina*^1^, Colin Mclean*^1^, Mike DR Croning^2^, Katharina F Heil^1,4^, Emilia Wysocka^1^, Xin He^5,6,1^, David Sterratt^1^, Seth GN Grant^2,5^, Thomas I Simpson^1,5^, and J Douglas Armstrong^1,3,5^.

Email: Colin Mclean* - [Colin.D.Mclean@ed.ac.uk](mailto:Colin.D.Mclean@ed.ac.uk) and Oksana Sorokina* - Oksana.Sorokina@ed.ac.uk

*Corresponding author

1. The School of Informatics, University of Edinburgh, Edinburgh, United Kingdom.

2. Centre for Clinical Brain Sciences, University of Edinburgh, Edinburgh, United Kingdom

3. Computational Biomedicine Institute (IAS-5 / INM-9), Forschungszentrum Jülich, Jülich, Germany

4. University of Barcelona, Barcelona, Spain

5. Simons Initiative for the Developing Brain, University of Edinburgh, Edinburgh, United Kingdom

6. Dementia Research Institute, University of Edinburgh

# Methods.

## Data collection and ID mapping.

We systematically curated synaptic proteomic datasets from the literature, to produce a comprehensive index of the proteins (and their genes) reported at the synapse. To find proteomic studies we searched PubMed using the key words: “synaptic proteome”, “postsynaptic proteome”, “presynaptic proteome”, “synaptosome”. Preference was given to the studies focussing on mammalian brains in healthy/normal experimental conditions. Thus, many genetic conditions, e.g. disease specific and sex-specific studies, identified where not included at that point. The last PubMed query was performed in September 1919. However one additional study was added after this point, Taoufiq et al., 2020, which we believe made the presynaptic dataset more complete. A total of 58 papers describing a landscape of 8087 synaptic genes (Table 1) were annotated with the following metadata: PUBMED ID, species, method of extraction, number of identified proteins and brain region. Each study’s respective protein list was extracted and mapped to stable identifiers (MGI, Entrez and Uniprot) for the predicted set of orthologues for three species (human, mouse, rat). Additional functional information (e.g. GO function, disease association) was overlaid as metadata onto the vertices*.* Supplementary Table 1 contains a detailed list of the papers found and associated metadata. It contains the information for PubMed ID and some useful metadata such as species (mouse, human, rat), protein counts, brain region where the sample was taken from along with experimental methods used for protein identification and quantification.

Protein/gene lists from the published synaptic proteome studies were combined into the total list of synaptic components. Identifiers from each study were mapped to stable IDs including: Entrez Human and Mouse, Uniprot and MGI IDs. The resulting 'master list' contains only unique gene entries (rows) with corresponding studies (columns) where they have been identified. For example, if a protein was discovered in several studies all those respective columns will contain 1. The protein overlap for synaptic compartments is shown at Figure 1, H in the main text and at the Figure 1 below.

*Figure 1. The overlap for synaptic compartment components obtained based on 58 studies.*

## Prediction of total size of pre- and postsynaptic proteome.

Only proteins that were found more than one time were taken into account to make the most confident “consensus” dataset for pre-and post-synaptic proteomes.

We fitted the accumulation of new proteins against the year they were first time identified in R*,* using linear (y ~ x), and non-linear (y ~SSlogis (x, Asym, xmid, scal)) models. The goodness of fit was compared by Akaike’s Information Criteria) AIC function [75], where lower indicates a more parsimonious model.

For post synaptic proteome the non-linear model is shown at Figure 1F and predicted maximum size of “consensus” PSP proteome is 3499, achieved by roughly 2023.

AIC coefficient is 213.8955 for linear fit and 205.0504 for non-linear fit, which means the latter is more parsimonious.

By AIC criteria, the linear model for presynaptic “consensus” proteome is better than non-linear (103.2001 and 107.2766, respectively), which likely means that presynaptic proteome is not in its “saturation” phase yet.

## PPI data.

We generated a Protein-Protein Interaction (PPI) dataset, based on three well established publicly available databases: DIP [62], IntAct [61](“psimitab/intact.zip” released on August 1^st^ 2016 and March 2^nd^ 2017) and BIOGRID [60](“BIOGRID-ALL-3.4.139.mitab.zip” respective versions, released in August 2016 and version 3.4.147, March 2017) was used.

The interaction data from each database was extracted in the PSI-MITAB format [73, 74]. To merge the multiple interaction datasets together we standardised the different interaction IDs used, by mapping each to Entrez gene IDs. This was achieved using a 'bimap' file created from the consolidation of mapping files collected from NCBI ftp server (<ftp://ftp.ncbi.nlm.nih.gov/gene/DATA/gene_refseq_uniprotkb_collab.gz>, <ftp://ftp.ncbi.nlm.nih.gov/gene/DATA/gene2accession.gz>; accessed: 2015-12-08) and id mapping file from UniProtKB (<ftp://ftp.uniprot.org/pub/databases/uniprot/current_release/knowledgebase/idmapping/idmapping_selected.tab.gz>; accessed: 2015-12-08). Furthermore additional information was checked and unified, e.g. different “MI-ID” tags were mapped to the same format “MI:identifier”. (Further details can be found in Supplementary Methods).

To obtain only direct interactions, the 'interaction type' column was then filtered for the “association” (MI:0914), “physical association” (MI:0915) and 'direct interaction' term (MI:0407) and its 63 child-terms. Some of the source data uses an obsolete interaction type MI:0218, “physical interaction” which was also included, since it was updated to association and physical association, which we both include. PPIs based on the interaction types: “genetic interaction” (MI:0208) (including “suppression” (MI:0796) and “synthetic” (MI:0794)), “colocalization” (MI:0403), “genetic interference” (MI:0254) and “additive genetic interaction defined by inequality” (obsolete term, MI:0799) were excluded from the final set.

To obtain the confident set of direct physical interactions we also excluded the predicted interactions and interactions obtained by Co-IP experiments (spoke models), filtering out the PSI-MI terms like “Pull-down”, “Affinity technology”, etc.

After filtering for direct interactions 126579 unique, direct human interactions were retrieved. For further analysis we were interested in the number of interactions in our datasets: the pre- and post-synaptic, and synaptosome datasets. Table 1 summarises the number of internal PPIs in each set respectively.

| Total N of unique Human PPIs extracted from three databases | N of unique Human PPIs after filtering for direct/physical interaction | N of unique PPIs in PSP network | N of unique PPIs in PSP “consensus” network | N of unique PPIs in Presynaptic network |
| --- | --- | --- | --- | --- |
| 407,643 | 126,579 | 28,915 | 14,496 | 8,678 |

Table 1. *Summary of protein-protein interactions (PPIs) in this study. PPI numbers before and after filtering for direct and physical interactions.*

## Clustering

Clustering, or community detection, in networks has been well studied in the field of statistical physics [1] with particular attention to methods developed for social science networks. The underlying assumption(s) of what makes a community in social science, translates remarkably well to what we think of as a community (sub-complex, module or cluster) in PPI networks. This has lead to the wide application of the unsupervised cluster detection measure Modularity (Q) [2] in social and biological network studies. Modularity can be naturally applied to our synaptic PPI networks, were we use Modularity to identify molecular clusters in our synaptic PPI networks, which maximise the PPIs within each cluster relative to PPIs between external clusters. We use a Spectral based Modularity algorithm [3] to reveal the community structure in our PPI networks. The result of applying the Spectral algorithm to the PSP network is 94 communities, with a Modularity value of 0.37, as illustrated in Figure 3a.

Without ground truth data to test community assignment against, we tested the stability of clusters founds on each network with a consensus based clustering approach [4, 5]. A consensus matrix for the Spectral algorithm on each network was built using the distributed computing facility provided by ECDF (Edinburgh Compute and Data Facility, U of Edinburgh. 2013, *www.ecdf.ed.ac.uk*) by randomly selecting 80% of the network genes to generate a clustering result and repeating 500 times. The consensus matrix measures the frequency that any pair of genes are likely to cluster together and was used to assign a probability to each gene belong to each observed community. The stability of each cluster was then measured from the mean gene probability using the gene’s observed community. Darker coloured communities are found more stable than lighter coloured communities, as illustrated for the PSP network in Figure 3, A.

| **Algorithm** | **Modularity (Q)** | **No: C** | **No: Cn=1** | **No: Cn>=100** | **Max (Cn)** | **Avg (Cn)** | **PAC** | **µ** |
| --- | --- | --- | --- | --- | --- | --- | --- | --- |
| **Spectral** | 0.37 | 94 | 32 | 14 | 453 | 51.2 | 8.41E-5 | 0.47 |

Table 2*.* *Cluster characteristics for the Spectral algorithm applied to the Largest Connected Component for the PSP PPI network (N:4817 E:27788). The maximum Modularity obtained, number of detected communities (C), the number of communities with size (Cn) equal to 1, the number of communities >= 100, the size of the largest community, the average community size. PAC value (at the 0.9 and 0.1 consensus interval limits) for the Spectral algorithm, obtained from the CDF of the consensus matrix, generated from 500 random sub-samples of 80% of the node size. Where the PAC score measures the proportion of ambiguously clustered pairs (PAC) [6]. And the mixing parameter, µ measures the fraction of edges lying between communities*

To further dissect the potential sharing of pathways between AD and HTN Gene Disease Annotations (GDA’s) in the PSP network (Figure 3, f), we employed a Belief Propagation algorithm, to propagate these GDA’s through the network’s edges, and a Degree-Corrected Block Model (DC-SBM) to model its effect on network clustering. The benefit of using such a model over the Spectral algorithm is that this model ‘a priori’ assumes no correlation between the GDA’s and the network communities and will uncover such a correlation only if one exists. We implemented, and ran, a parallel version of the code supplied in [7] (<https://github.com/cmclean5/rblock>). We set the number of blocks in the model to 46, using the clustering results from the Spectral algorithm as a guide: 46 being the number of communities with node size greater or equal to 5. We ran the algorithm 10 times, each time randomising the initial values of parameters, and selected the run with the highest likelihood value.

## Estimation of Bridgeness and semi- local centrality measures.

To assess the topological importance of the proteins in PSP network we estimated two independent measures for each protein.

1) semi-local centrality Cl(v), which takes into consideration both a vertex’s degree, its nearest, and next to nearest neighbours:

$$Q\left( u \right)=\sum_{w\in\Gamma\left( u \right)} N\left( w \right)$$

$Cl\left( v \right)=\sum_{u\in\Gamma\left( u \right)} Q\left( u \right)$

- (1)

where $\Gamma\left( u \right)$ is the set of nearest neighbours of *u* and *N(w)* is the number of nearest and next to nearest neighbours of the vertex *w*. We performed unity-based or feature scaling: X – Xmin/(Xmax-Xmin) to normalise the semi-local centrality to lie in the range [0,1].

Semi-local centrality differs from degree centrality therefore, in making use of more information, allowing us to also measure a vertices' spread' of information locally through the network.

2) Bridgeness *B(v)* of vertex *v* to measure the influence of a gene due to the clustering [64] can be estimated as:

$B\left( v \right)=1-\sqrt{\frac{c}{c-1}{\sum_{j=1}^{c} \left( u_{jv}-\frac{1}{c} \right)}^{2}}$-(2)

Where $u_{v}$ is the community membership vector for vertex *v,* which is the probability of vertex v belonging to a given community $u_{v}=\left[ u_{1v},u_{2v},\ldots,u_{cv} \right]$,

where $\sum_{v.j} u_{jv}=1$ and c is the number of communities detected by the algorithm.

The combination of these measures allows categorisation of the influence each has on the overall network structure (Figure 3):

## Graph entropy and perturbations.

We tested for evidence of structure in each network by performing a entropy based perturbation analysis. In this analysis the global entropy rate (SR) of the network is measured after each gene is perturbed, either by over-expression (SR_UP) or under-expression (SR_DOWN) and plotted against the degree of the perturbed gene. To assign expression values to each protein in the perturbation analysis we followed [8]. Proteins were set to initial values of 2 with perturbed values of 14 when modelling actively and set to initial values of 16 with perturbed values of – 14 when modelling inactively. For each PPI network we observed a bi-modal response between gene over-expression and degree and opposing bi-phasic response relative to over/under-expression between global entropy rate and degree. This is illustrated in Figure 3b for the PSP network, where in this figure the dashed line shows the initial global entropy rate (SRo = 0.668) for the unperturbed network. This type of bi-modal, bi-phasic behaviour has been observed only in networks with scale-free or approximate scale-free topology [8].

Since lower entropy systems implies more order, we also used graph entropy to test for structure in the PSP network against two randomised network models: the Eros-Renyi (E-R) model, and one which follows a scale-free (Power-Law) degree distribution (P-L) [9, 10]. To generate randomised networks models we made use of the R package 'igraph' [11](version 1.0.0), and used the same number of nodes and edges found in the observed PSP network, and the power-law exponent obtained from fitting the PSP degree distribution (i.e. α = 2.41) for the scale-free random model. From 1000 randomly generated networks, we measured the global entropy rate (of the unperturbed network) for the Erdos-Renyi (E-R=0.9890+-0.0005) and Power-Law (P-L=0.9127+-0.0032) models to be much high than that of the observed PSP network (SRo = 0.668). The difference in graph entropy, between the observed network and randomised models, indicating the connections found in the PSP network are not random.

## Disease Network Localisation.

We compared the gene-disease association for the pre and post-synaptic PPI networks using the combined OMIM/GeneRIF/Ensembl variation data for common set of synaptic diseases (or synaptopathies): Schizophrenia (SCH), Autistic Spectrum Disorder (ASD), Autistic Disorder (AUT), Bipolar Disorder (BD), Intellectual Disability (ID), Alzheimer disease (AD), Epilepsy Syndrome (Epi), Parkingson's Disease (PD), Frontotemporal Dementia (FTD), Huntington's Disease (HD), Multiple Sclerosis (MS) and Hypertension (HTN)

We investigated the overlap and separation of each disease-disease pair by measuring the mean shortest distance for each disease, using the shortest distance between each GDA to its next nearest GDA neighbour[12]. The overlap, or separation, of each disease-disease pair in the pre- post-synaptic PPI networks, could then be quantified using:

$$S_{AB}\equiv\left\langle d_{AB} \right\rangle-\frac{\left\langle d_{AA} \right\rangle+\left\langle d_{BB} \right\rangle}{2}$$

- (3)

Where $\left\langle d_{AA} \right\rangle$and $\left\langle d_{BB} \right\rangle$quantify the mean shortest network distance between genes associated with disease A (or B), and $\left\langle d_{AB} \right\rangle$the mean shortest distance between diseases. $S_{AB}$ is bound by the diameter of the network, i.e., $d_{max}\leq S_{AB}\leq d_{max}$where $d_{max}$is 8, 7, 8 for the presynaptic, PSP and PSP consensus PPI networks respectively. The magnitude of $S_{AB}$depends on the number of GDSs associated with each disease. Large positive values imply two well separated diseases, while large negative values indicate large (number of GDAs) diseases with a big overlap, often implying one disease is the variant or precursor to the other. Each disease-disease network separation pair ($S_{AB}$) was compared against a full randomised model: drawing the same number of GDAs (from the set of all network genes) for each disease at random, before computing its separation. For each disease-disease pair, we performed 10,000 iterations of the full randomised model using the ECDF distributed computing facility.

The difference between the observed and randomised disease pair separations, was quantified using the z-score:

$$z-{score}_{AB}=\frac{S_{AB}-\left\langle S_{AB}^{rand} \right\rangle}{\sigma\left( S_{AB}^{rand} \right)}$$

- (4)

Where $\left\langle S_{AB}^{rand} \right\rangle$and $\sigma\left( S_{AB}^{rand} \right)$are the mean and standard deviation obtained from the 1000 iterations. Each disease-disease pair separation using the full randomised model, i.e., $S_{AB}^{rand}$, was found to follow a normal distribution. We therefore assessed the significance of each disease-disease pair's separation, from P-values estimated from its z-score calculated in (4):

$P-value\left( X=-\left| Z-{score}_{AB} \right|;\mu=0,\sigma=1 \right)=\left[ 1+erf\left( \frac{x-\mu}{\sigma\sqrt{2}} \right) \right]$- (5)

Where we take the negative of the absolute value of each disease-disease pairs z-score calculated in (4) and make use of R's *pnorm* function available in the 'stats' package (R version 3.4.2).

The confidence in each disease-disease pairs P-value was tested for by calculating its q-value [76], and from the Bonferroni correction at the 0.05 (*), 0.01 (**) and 0.001 (***) significant levels.

## SQLite database.

*Figure 2. Database structure. The database is provided as a Supplementary File with a screencast for use case examples. An Rmd file is provided for querying database under the R environment.*

The database contains the following main tables:

- Gene: list of genes including IDs (MGI, Entrez Human and Mouse) and gene names (Human and Mouse).
- Specie: Tax ID (Human and Mouse)
- Paper: list of papers with PMID ID, name (in format “FirstAuthor_year”), year of publication
- Location: postsynaptic, presynaptic, synaptosome
- Method: shotgun or IP
- Brain region: list of regions where the samples originate, with hierarchical region structure
- PPI: human protein-protein interactions combined from BioGRID, Intact and DIP databases, contain information on methods, interaction type (PSI-MI nomenclature) and PMID info for each of the interactions.
- PaperGene: table links gene to respective papers and the metadata above
- GO: BP, CC and MF GO annotation for Human, Mouse and Rat species
- GOGene: gene to GO association list
- Disease: List of diseases from HDO for Human
- DiseaseGene: genes to disease association list
- GeneToModel: genes with found association with published model of synaptic plasticity

The database is created with SQLite v 3.31.1 RDBMS in SQLite Studio v3.2.1.

The database is easily accessible from SQLite Studio (the separate manual and screencast provided). The database is accessible from RStudio (Rmd provided, you just need specify the path to the database).

## Code and extending the database.

All the raw data tables and R code that generates the SQLite database from them are freely available from GitHub https://github.com/oksankas/Synaptic-proteome-DB.

The easiest way to integrate your own dataset would be:

1) select appropriate localisation table from Presynaptic (“Pres_DB_April21.txt”), Postsynaptic (“PSD_db_Oct20.txt”), or Synaptosome (“Syn_DB_April21.txt”) and add a new column in a way similar to previously collected studies.

2) Edit table “Full_DB_April21” with the new genes, if any.

3) Edit the table Paper_Summary_April21” to add your new study.

4) Run the R code Populate_DB_April21 to rebuild the database from scratch. Make sure you provide right paths to the tables.

References.

1. Fortunato, S., *Community detection in graphs.* Physics Reports, 2010. **486**: p. 75-174.

2. Newman, M.E., *Modularity and community structure in networks.* Proc Natl Acad Sci U S A, 2006. **103**(23): p. 8577-82.

3. McLean, C., et al., *mproved Functional Enrichment Analysis of Biological Networks using Scalable Modularity Based Clustering.* Journal of Proteomics and Bioinformatics, 2016. **9**(1): p. 9-18.

4. Stefano, M., et al., *Consensus Clustering: A Resampling-Based Method for Class Discovery and Visualization of Gene Expression Microarray Data.* Mashine Learning, 2003. **5291-118**.

5. Simpson, T.I., J.D. Armstrong, and A.P. Jarman, *Merged consensus clustering to assess and improve class discovery with microarray data.* BMC Bioinformatics, 2010. **11**: p. 590.

6. Șenbabaoğlu, Y., G. Michailidis, and J.Z. Li, *Critical limitations of consensus clustering in class discovery.* Sci Rep, 2014. **4**: p. 6207.

7. Newman, M.E. and A. Clauset, *Structure and inference in annotated networks.* Nat Commun, 2016. **7**: p. 11863.

8. Teschendorff, A.E., et al., *Increased signaling entropy in cancer requires the scale-free property of protein interaction networks.* Sci Rep, 2015. **5**: p. 9646.

9. Fan, C. and L. Linyuan, *Connected Components in Random Graphs with Given Expected Degree Sequences.* Annals of Combinatorics, 2002. **6**: p. 125-145.

10. Cho, Y.S., et al., *Percolation transitions in scale-free networks under the Achlioptas process.* Phys Rev Lett, 2009. **103**(13): p. 135702.

11. Gabor, C. and N. Tamas, *The igraph software package for complex network research.* InterJournal, 2006. **Complex Systems**: p. 1695.

12. Menche, J., et al., *Disease networks. Uncovering disease-disease relationships through the incomplete interactome.* Science, 2015. **347**(6224): p. 1257601.
